# Experimental and Numerical Investigation of Flow and Alignment Behavior of Waste Tire-Derived Graphene Nanoplatelets in PA66 Matrix during Melt-Mixing and Injection

**DOI:** 10.3390/polym13060949

**Published:** 2021-03-19

**Authors:** Kuray Dericiler, Hadi Mohammadjafari Sadeghi, Yavuz Emre Yagci, Hatice S. Sas, Burcu Saner Okan

**Affiliations:** 1 Integrated Manufacturing Technologies Research and Application Center & Composite Technologies Center of Excellence, Manufacturing Technologies, Sabanci University, 34906 Istanbul, Turkey; kuraydericiler@sabanciuniv.edu (K.D.); mhadi@sabanciuniv.edu (H.M.S.); 2 Faculty of Engineering and Natural Sciences, Sabanci University, Tuzla, 34956 Istanbul, Turkey; 3 Farplas Otomotiv A.S., Taysad Organize Sanayi Bölgesi (TOSB), 41420 Kocaeli, Turkey; e.yagci@farplas.com

**Keywords:** graphene, waste materials, upcycling, polymer nanocomposites, modeling, simulation

## Abstract

Homogeneous dispersion of graphene into thermoplastic polymer matrices during melt-mixing is still challenging due to its agglomeration and weak interfacial interactions with the selected polymer matrix. In this study, an ideal dispersion of graphene within the PA66 matrix was achieved under high shear rates by thermokinetic mixing. The flow direction of graphene was monitored by the developed numerical methodology with a combination of its rheological behaviors. Graphene nanoplatelets (GNP) produced from waste-tire by upcycling and recycling techniques having high oxygen surface functional groups were used to increase the compatibility with PA66 chains. This study revealed that GNP addition increased the crystallization temperature of nanocomposites since it acted as both a nucleating and reinforcing agent. Tensile strength and modulus of PA66 nanocomposites were improved at 30% and 42%, respectively, by the addition of 0.3 wt% GNP. Flexural strength and modulus were reached at 20% and 43%, respectively. In addition, the flow model, which simulates the injection molding process of PA66 resin with different GNP loadings considering the rheological behavior and alignment characteristics of GNP, served as a tool to describe the mechanical performance of these developed GNP based nanocomposites.

## 1. Introduction

Non-aromatic polyamides (PAs) are widely used as engineering thermoplastics in various fields such as aerospace, food packaging, and especially preferred in the automotive industry as engine compartments, bearings, oil pans, and various under-the-hood parts [1] due to their easy processability, good thermal stability, favorable price range, relatively high mechanical properties, superior wear resistance, and low density [2]. Among PAs, PA66 has taken great attention due to less water and moisture absorption than PA6, since moisture uptake affects the composites’ mechanical performance and rheological properties adversely. In other words, the humidity absorption and low dimensional stability of PAs caused by intrinsic hydrophilic amide groups, along with their sensitivity to shock and relatively low impact resistance, limits their usage [3,4].

In the literature, numerous studies have been reported on the enhancement of mechanical properties of PA-based composites by compounding with micro/nano additives such as montmorillonite [5], SiO_2_ [6], and carbon-based materials such as carbon black [7], multi-walled carbon nanotubes [8], and exfoliated graphite [9] as well as by blending with other polymers such as high-density polyethylene [10], and polyphenylene sulfide [11]. However, high anisotropy and aggregation of carbon nanotubes (CNTs), stacking problems of exfoliated graphite, and brittleness in the presence of carbon black do not meet the requirements in the compounding process. Meanwhile, in recent years graphene is a prominent alternative to micron/nano additives due to its outstanding mechanical, thermal, and electrical properties and its 2D structure that provides ease of dispersion in the polymer matrix [12].

Several methodologies have been proposed for the effective incorporation of graphene and its derivatives as the main reinforcement into polyamide-based composites by in situ polymerization, solution mixing, and melt compounding. Fu et al. distributed few-layer graphene powders in PA6 matrix by in situ polymerization and achieved an increase of 13%, 44%, and 47% in the tensile strength, flexural strength, and impact strength, respectively, with 0.5 wt% loadings [13]. Li et al. fabricated foam-like structures from graphene oxide by hydrothermal method then utilized in situ polymerization of polymeric precursors to obtain 3D-Graphene/PA6 nanocomposite, which resulted in an increase of 400% in the thermal conductivity of composite at 2 wt% loading as well as improved anti-dripping properties for heat-retardance applications [14]. Besides, Duan et al. used graphite oxide powder and in situ polymerization method to produce reduced graphene oxide (rGO)/PA66 nanocomposites, and with 0.75 wt% loadings, they obtained 9% and 6% increase in yield strength and Young’s modulus, respectively, while maintaining the impact strength [15]. Although the in-situ polymerization technique promotes good dispersion of graphene in the polymer, its adaptation to produce nano-integrated compounds at the industrial amounts has limitations due to scale-up inefficiency and economic issues.

There are several challenges in integrating graphene and its derivatives as the main reinforcement into the polymer matrix. Poor dispersion of graphene in the polymer causes agglomeration of the 2D layers and induces weak interfacial connection, which hinders obtaining theoretical superior composite properties. In order to overcome this problem, researchers have made modifications to the graphene and polymer matrix. For instance, Sarno et al. described a supercritical CO_2_-assisted process to develop GO-loaded polymer membrane supercapacitors [16]. The authors indicated that the supercritical process aided to prevent agglomeration of GO particles even at 90% loading for the aerogels. In another study, Scaffaro et al. utilized modified Tour’s method [17] to obtain graphene oxide (GO), then modified graphene oxide with nanosilica (GOS) and fabricated GOS/PA6 composites by batch compounding method, which yielded 180% and 210% increase in Young’s modulus and tensile strength at 0.5 wt% GOS loading, respectively [18]. Moreover, Wang et al. exfoliated graphene sheets in the liquid phase and modified them with tannic acid to fabricate tannic acid-modified graphene sheets (TAGS)/PA66 composite by solution mixing and drying method and obtained 15.9% and 118% improvement in Young’s modulus and tensile strength at 0.5 wt% TAGS loading, respectively [19]. In another study, Cai et al. prepared graphene oxide by Hummers’ method [20], mixed GO with PA1212 in the liquid phase, dried, and melt compounded to fabricate the composite in a two-step manner, which demonstrated an improvement of 10% in yield strength at 0.7 wt% loading [21]. Furthermore, Gong et al. prepared GO by modified Hummers’ method, modified the GO with polyvinyl alcohol (PVA) and fabricated PVA modified GO/PA6 composites by solution mixing technique, obtaining 34% and 41% increase in yield strength and Young’s modulus, respectively, for 2 wt% GO loading [22]. Therefore, it is critical to employ some form of modification to improve the interfacial connection between graphene and the polymer interface.

In addition to graphene utilization as the main reinforcing agent, graphene carries a significant potential for co-reinforcement with commercial reinforcements such as glass fiber (GF) in the compounding process. In one of the studies, Pan et al. investigated the effect of graphene nanosheets (GNS) produced by Hummers’ method and hydrazine reduction on the fire and mechanical properties of glass fiber PA6 composites having aluminum hypophosphite resulting in the improvement of bending strength by 44% at the cost of a decrease in the tensile strength by 38% by the addition of 1 wt% GNS [23]. Cho et al. functionalized commercial GO with acyl chloride (AGO) and utilized them together with CNT and in situ polymerization of PA66 to coat carbon fibers (CF), resulting in the improvement of interfacial shear strength and tensile strength by 160% and 136%, respectively, at 1 mg AGO and 0.5 mg CNT [24]. Karatas et al. melt compounded commercial graphene nanoplatelets (GNP) together with CF and PA66, which yielded a 57% reduction in adhesive wear and 11% improvement in tensile strength with the addition of 0.5 wt% GNP [25]. Moreover, in our previous study, the addition of GNP from recycling and upcycling waste tire to the PA66 and compounding them with GF demonstrated 23% improvement in the flexural modulus [26] at 1% GNP loading. To conclude, a synergistic effect of graphene with hybrid composites with GF and CF yields significant improvements in mechanical and tribological properties.

There are challenges in the integration of graphene and its derivatives into a polymer matrix, especially in melt-phase due to their weak interfacial connections between the main reinforcement, inhomogeneous dispersion, and lacking proper functional groups [18,19,21,22,27]. Rheology is reported as a strong phenomenon to comprehend the reinforcement’s dispersion characteristics in the polymer, which depends on the size, shape, and concentration of the graphene and the interaction between the graphene and the polyamide [28]. In one of the studies, Mayoral et al. demonstrated that at low frequencies, neat PA6 exhibits viscous behavior, whereas, at high frequencies, polymer chain entanglements dominate rheological response; however, with the addition of 15% GNP at 240 °C and 1% strain, rheological percolation is achieved, and GNPs start to agglomerate which degrades mechanical properties of melt-mixed GNP/PA6 composite [29]. Furthermore, Pan et al. linked the melt mixed GFPA6 composite’s flame resistance property containing aluminum hypophosphite and GNS with the viscosity at 100 rpm and 215 °C. The authors demonstrated that the increase in the torque yields improved viscosity related to anti-dripping behavior for up to 2% GNS loadings [23]. In another study, Canales et al. developed a new rheological model based on solid content in the molten mix and showed that the crystallization process is accelerated considerably, even with the low amounts of graphene concentration dispersed in the “amorphous” PA6 matrix. This valuable outcome reports that the crystallization time and crystallization degree of a polymer system favorably affect mechanical performance [30]. Thus, it is critical to understand the polymer system’s rheological behavior to implement the required reinforcements further to achieve desired performance improvements.

The graphene nanoplatelets will orient during the injection process, and the flow characteristics such as shear rate and injection pressure will determine the final orientation pattern. The ultimate pattern of the GNPs can be incorporated with the sample’s physical characteristics, such as mechanical and thermal properties. Throughout the injection process, fluid flow and GNPs’ rotational and transitional motion interrelate with each other. The rheology of flow with microstructure suspensions has been studied experimentally for Newtonian and non-Newtonian fluids [31]. It has been shown that the presence of the microstructure suspensions changes the shear and free shear flows in Newtonian and non-Newtonian fluids [32,33]. The prediction of the behavior of the fiber/plate shape suspension flows requires a more compressive approach than the sphere shape suspension flow due to their anisotropic shape. According to the literature, the fiber suspension is categorized by their aspect ratio into short or long suspension so that microstructures with aspect ratios smaller than 100 are considered short suspension fibers [34]. The existing forces governing the flow in the system can be hydrodynamically orientated and also includes the fiber–fiber interaction forces. Increasing the number of suspended particles will increase the fiber–fiber interaction forces, while in the fewer number of suspensions, the fibers can easily move without any interaction forces. This regime can be assumed when the number of fibers per unit volume n is much smaller than 1/L^3^, where L is the average length of the particles in the suspension. Thus, understating the behavior of the flow of GNPs suspension will enable the prediction of their distribution, which affects the sample’s physical characteristics [35].

Melt-compounding techniques for the formulation development of graphene-based composites carry a significant potential to initiate graphene commercialization in engineering plastics due to its ease of production and economic feasibility. In one of the studies, Cho et al. investigated the addition of rGO produced by modified Hummers’ method followed by hydrazine reduction under microwave treatment, and modified by titanate coupling agent and improved the thermal conductivity of PA by 53% with 5 wt% loading [36]. In another work, Karatas et al. utilized commercial GNP to produce GNP/PA66 by melt compounding and obtained significantly lower friction coefficients while increasing the tensile strength by 22% at 0.5 wt% GNP loading [25]. However, getting useful dispersions in the melt compounding process is challenging and often requires compatibilizer or surface modification to provide a good reinforcement/matrix connection.

In the present study, waste tire-derived GNPs were distributed in the PA66 matrix by the thermokinetic mixer in the melt phase by controlling GNP ratios and decreasing the loading ratios down 0.5 wt%. Experimental studies were supported by numerical work to understand the interfacial interactions between GNP and PA66 polymer chains and also the alignment of platelet structures in the PA66 matrix during melt mixing and injection molding. The detailed characterization was carried out to monitor GNP’s effect on the crystallinity and rheological properties of PA66 based nanocomposites. A numerical study provided the necessary process modeling data to give insight into the injection molding process. Previous reports include using experimental data to predict rheological properties [37], compression molding [38], and aging behavior [39] of neat PA66, as well as non-linear damage model [40] and low-velocity impact behavior [41] of glass fiber reinforced PA66. However, to the best of our knowledge, this is the first study to link numerical data to experimental work for investigating the injection molding behavior of GNPs in the PA66 matrix as well as their effect of orientation on the mechanical and rheological properties of PA66/GNP composites. Successfully modeling the process dynamics of PA66 and its GNP-based nanocomposites will offer a significant advantage to understand the interfacial interaction between polymer chains and nano/hybrid additives during extrusion and injection processes.

## 2. Materials and Methods

### 2.1. Materials

Graphene nanoplatelets (GNP) derived from waste tires by recycling and upcycling processes were obtained from Nanografen Co., Istanbul, Turkey. The density of the waste tire-derived GNPs is calculated as 2.115 g/cm^3^. Other characterization results of GNP in terms of XPS, Raman spectroscopy and TEM is given in Figure S1 (see supporting information). Additionally, XRD pattern of the GNPs are also given in Figure S2 (see supporting information). Polyamide 66 (EP 158, Ravago, Istanbul, Turkey) is a general-purpose polymer with a melt flow rate of 71 g/10 min, good toughness, and high impact properties. PA66 was dried in the oven at 80 °C before each usage to remove the moisture. The characteristic properties of GNPs have been provided in supporting information.

### 2.2. Fabrication of GNP Reinforced PA66 Nanocomposites by Thermokinetic Mixer

GNP reinforced PA66 composites were prepared by a custom-made Gelimat thermokinetic mixer at a shear rate of ~4700 rpm at 300 °C for 1 min. The loading ratios of 0.2, 0.3, 0.4, 0.5, and 1.0 wt% GNP were adjusted and mixed with PA66 at the melt-phase to attain homogeneous dispersion and provide a high degree of exfoliation through polymer chains. The obtained products were then crushed to granules and injection molded by an mini-injection molding machine (Xplore, Sittard, The Netherlands) for mechanical tests.

### 2.3. Characterization

Characteristic properties of graphene and its nanocomposites were examined by using various spectroscopic and microscopic techniques. Thermal analysis of polymer composite samples was carried out by Differential Scanning Calorimetry (DSC) method using DSC 3 + 700 (Mettler Toledo, Columbus, OH, USA) under the nitrogen atmosphere from 25 °C to 300 °C. Dried samples were heated and held at 300 °C for 5 min to eliminate the thermal history. An additional heating cycle and cooling cycle were performed at the rate of 10 °C/min to investigate the melting properties, thermal behavior, and crystallization degrees of the samples. STARe software (Mettler Toledo, Columbus, OH, USA) was used in order to obtain the melting enthalpy (ΔHm), crystallization enthalpy (ΔHc) as well as melting (Tm), and crystallization temperatures (Tc). In order to carry out the morphological studies, mechanical test specimens were fractured in liquid nitrogen and coated with a thin layer of gold. Surface topography and morphology were investigated using a Leo Supra 35VP Field Emission Scanning Electron Microscope (FESEM, Carl Zeiss AG, Jena, Germany). Crystalline structures of the samples were studied by X-ray Diffraction (XRD) method using a D2 PHASER Desktop diffractometer (Bruker, Billerica, MA, USA) utilizing a CuKα radiation source. Elemental analysis has been carried out using X-ray Photoelectron Spectroscopy (XPS, Thermo Scientific, Waltham, MA, USA) for graphene samples. The mechanical tests were conducted by using 5982 Static Universal Test Machine (UTM, Instron, Norwood, MA, USA) with 5 kN load cell for ISO 527-2 tensile and ISO 178 three-point bending tests. MCR 702 TwinDrive Rheometer (Anton Paar, Graz, Austria) was used for the rheological characterization of the specimens.

### 2.4. Modeling Tools

A numerical model was developed for each case to examine additive GNP suspension flows through 2-dimensional sample geometries. The computational domains were considered based on the standard tensile and bending test sample geometries, which had an entrance diameter of 4 mm from the bottom boundary. An incompressible Newtonian suspending fluid was considered for the constitutive relationship, and simulations were performed with coupling the flow and equations for GNP orientation. In this study, the flow regime was laminar (see [42,43]), and the governing equations of continuity, momentum were given by:

(1) ∇.v=0

(2) ∇.σ=0

(3) $$\sigma =-pI+\mu \left[\left(\nabla v\right)+{\left(\nabla v\right)}^{T}\right]$$

where *v* is the velocity vector, *p* and *µ* is the pressure and viscosity, respectively. An implicit Backward Difference Method is applied to solve this problem. Equations were discretized using second-order triangular elements for velocity components, linear elements for pressure, and quadratic discretization for Stabilized Convection-Diffusion Equation.

GNPs orientation in the polymer melt was affected by the flow field, which was defined by the melt flow geometry, the interaction coefficient, and particle kinematics [43,44]. In order to capture the GNPs’ kinematic, a second-order fiber orientation tensor was defined as below:

(4) $$a_{2}=\int \;\vec{p}\vec{p}\psi \;\left(\vec{p},t\right)d\vec{p}$$

where $\vec{p}$ is the unit vector and its axis was parallel to the axis of the GNP, and the integration was done over all the possible ranges of angles for orientation of GNP. The *a*_2_ can be demonstrated like the below tensor in the Cartesian coordination:

(5) $$a_{2}=\left[\begin{array}{ccc} a_{xx} & a_{xy} & a_{xz}\\ a_{yx} & a_{yy} & a_{yz}\\ a_{zx} & a_{zy} & a_{zz} \end{array}\right]$$

This tensor is symmetric and satisfies the normalization conditions for second-order tensor, which means that $a_{xx}+a_{yy}+a_{zz}=1$. Increasing the tensor’s order will improve the accuracy of the simulation to predict the fiber orientation state [45]. The evolution equation for *a*_2_, according to Folgar and Tucker, can be expressed by the following equation [46,47]:

(6) $$\frac{\partial }{\partial t}a_{2}+\;\vec{u}.\nabla a_{2}=\Omega .a_{2}-a_{2}.\Omega +\lambda \left(\dot{\varepsilon .}a_{2}+a_{2}.\dot{\varepsilon }-2\dot{\varepsilon }:a_{4}\right)+2C_{I}\dot{\varepsilon }\left(I-3a_{2}\right)$$

here *I* is the identity matrix, ∇ is gradient vector, Ω is vorticity tensor, ε is strain rate tensor, and *C_I_* is the interaction coefficient and is a parameter measured through empirical observation [48]. For this simulation, the particle orientation was examined in the Newtonian fluid, and the interaction coefficient required to stabilize the simulation was kept constant over the various simulations. The particles were also assumed in a rigid shape with an aspect ratio of L/D = 1/50, where L and D represent the average length and the average particles’ diameter, respectively. The initial length of the particles was defined as 0.001 mm. Other properties of the particles were adapted from experimental studies. Fluid viscosity was calculated from the experimental data and imported into the COMSOL software (Comsol, Burlington, MA, USA). The fourth-order orientation tensor’s components in the above equations need to be related to the second-order orientation tensor’s components. Thus, several closure approximations such as quadratic closure, invariant-based optimal fitting closure, hybrid closure, etc., had been introduced and discussed in the literature [42,43,46,48,49,50]. For the present work, the quadratic closure was applied where the high order orientation tensor a_4_ is equivalent to the dyadic product of the two second-order tensors, which can be written as $a_{4}=\;a_{2}a_{2}$. There are different types of closure approximation and quantifying their accuracy is problematic—this approximation provides a rough calculation for the particle orientation states. However, the purpose of this paper is not to estimate the GNPs orientations accurately, and it can give an overview of how the particles are relocated and how their movement is related to the results observed in the experiments.

The appropriate boundary conditions were applied to mimic the processing conditions. The pressure inlet with a maximum of 1.9 MPa was introduced to the injection boundary, and the normal stress was assumed to be zero at the outlet boundary to simulate the experimental condition. For the walls, the no-slip boundary condition is employed. The Dirichlet condition is applied to the entrance boundary in solving the partial differential equation for the evolution equation so that the isotropic distribution at the inlet boundary is provided.

As the simulation tool of the above equations, COMSOL Multiphysics 5.5 is adapted, where the laminar flow and three distinctive partial differential equations (PDE) for orientation were coupled together. The PDE equations were defined in the forms of convection-diffusion type, which has the following format, for instance, for a dependent variable $a_{xx}$:

(7) $$\frac{\partial a_{xx}}{\partial t}+\nabla .\left(-c_{num}\nabla a_{xx}\right)+\beta .\nabla a_{xx}=f$$

where $c_{num}$ is the numerical diffusion coefficient, and is required to have a stabilized solution, f is the source and is the right-hand side in the evolution equation for $a_{xx}$ and β is the convection coefficient, which is the velocity vector in this case. 

Additionally, computational domain shape, the generated mesh, and mesh and time sensitivity test had been provided in the supporting document. Heterogeneous triangular meshes for two types of the flow domain are given in Figure S3. The comparison between the different time steps and mesh size is also given in Figure S4. The numerical results were discussed in terms of injection velocity profiles, pressure profile, and GNP orientation.

## 3. Results and Discussion

### 3.1. Mechanical Performance of GNP Reinforced PA66 Nanocomposites

In the present work, GNP obtained from waste tire used as reinforcement has 9 wt% surface oxygen groups with a surface area of 130 m^2^/g and a platelet size of 50 nm. The characterization details of GNP are given in the supporting document. Intrinsic oxygenated groups on the surface of GNPs coming from its manufacturing process provides enhanced interfacial interactions with the amino groups in PA66 chains. This interfacial interaction allows the effective load transfer from matrix to strong GNP particles. In our previous work [51], GNP loading ratios were adjusted between 0.5–2.0 wt%, and significant improvement in both flexural and tensile properties was observed; however, there is a decreasing trend observed by increasing GNP amount higher than 2.0 wt%. In order to understand the reinforcement and nucleating effects of GNP at low loadings, the GNP amount was decreased down to 0.2 wt% in the current study. Therefore, GNPs with the loadings of 0.2–1.0 wt% were mixed with PA66 by a high shear thermokinetic mixer. Herein, the thermokinetic mixer provides to increase intercalation of polymer chains through graphene layers leading to the homogeneous dispersion of graphene in the polymer matrix. In order to obtain better dispersion and to determine the optimum loading amount, the tensile and flexural properties of GNP/PA66 nanocomposites were characterized in detail.

**Figure 1:**
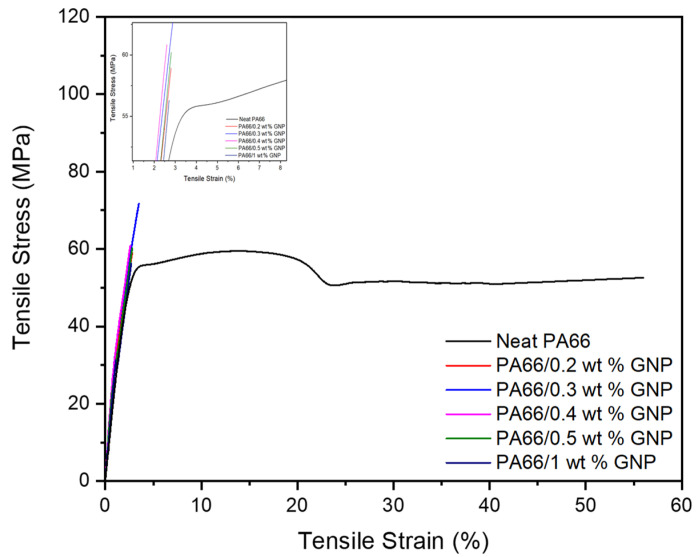
Tensile stress-strain curves of neat PA66 and PA66/GNP nanocomposites.

Figure 1 displays the tensile stress/strain curves of neat PA66 and its nanocomposites with different GNP concentrations. By adding GNP in the PA66 matrix, stiffness increased significantly compared to neat PA66 showing elongated characteristics. Table 1 summarizes the improvements by percentages of tensile strength and tensile modulus and tensile strain at break compared to the neat counterpart. According to tensile test results, the highest tensile strength improvement was obtained as 30.4% with 0.3 wt% GNP loading, and the highest tensile modulus was provided by incorporating 0.4 wt% GNP, which is similar to one having 0.3 wt% GNP. As the GNP amount was increased up to 1 wt%, there was a decreasing trend in the tensile strength. In other words, at higher loadings, the agglomeration of GNP reduces the interfacial area and creates stress concentration sites leading to an overall decrease in tensile properties [52]. In addition, as polymers’ mechanical properties are highly dependent on crystallinity [53], the improvement in tensile properties is a consequence of increased crystallinity, which is also confirmed by DSC analysis in the next section.

**Figure 2:**
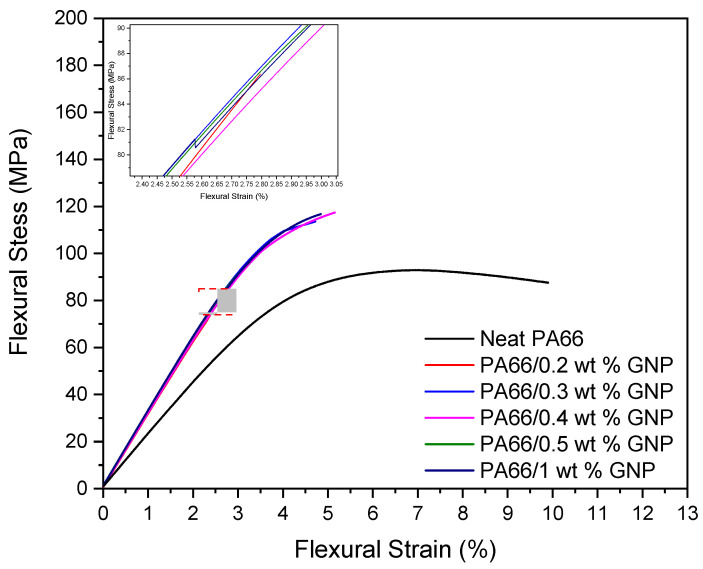
Flexural stress/strain curves of neat PA66 and its nanocomposites with different GNP loadings.

Figure 2 shows the flexural stress/strain curves of neat PA66 and its nanocomposites with different loadings after ISO 178 three-point bending tests. Flexural strength gives insight into a material’s resistance to fracture, while flexural modulus indicates a material’s tendency to bend [54]. It can be seen that the addition of GNP increased the flexural modulus with the increase of GNP loadings and also improved flexural strength except for 0.2 wt% loading. The highest flexural strength value was obtained by incorporating 0.4 wt% GNP as 114 MPa, which resulted in 22.4% improvement compared to neat polymer. Moreover, the addition of 0.3 wt% GNP achieved a 21.3% improvement in flexural strength as well as improved tensile strength. It is also worth noting that the flexural moduli have improved at least 40% in each GNP loading. This increase in flexural properties is led by graphene nanoplatelets acting as a nucleating agent in the polymer matrix. The early crystallization led to a higher degree of crystallinity, which subsequently improved the mechanical properties, as confirmed by DSC analysis later on. Table 2 summarizes the improvement percentages of flexural strength, strain, and modulus of the prepared composite specimens.

### 3.2. Thermal and Crystallinity Properties of GNP Based PA66 Nanocomposites

**Figure 3:**
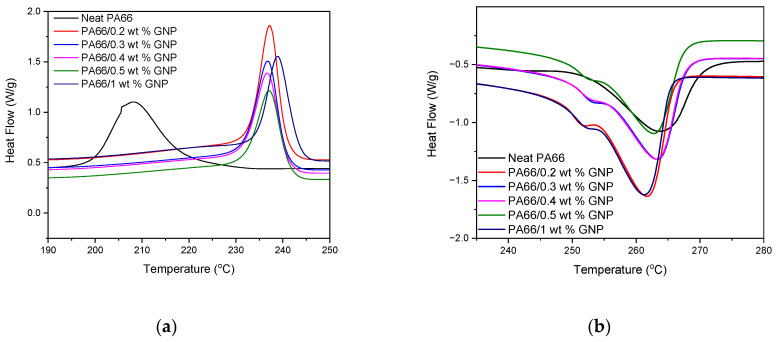
First cooling cycle (**a**) and second heating cycle (**b**) thermograms of PA66 and GNP-based PA66 nanocomposites.

Thermal properties of neat PA66 and GNP-based composites were investigated using the DSC method, which gives insight into the crystallization and melting behavior of the composites. The first cooling cycle for crystallization behavior and the second heating cycle melting curves of neat PA66 and PA66-GNP composites have been shown in Figure 3a,b, respectively. Thermal parameters have been summarized in Table 3. DSC analysis shows no significant changes for the melting behavior of neat PA66 and GNP-PA66 composites; however, the crystallization temperature increase indicates that GNP in the polymer acts as a nucleating agent and initiated early crystallization. As crystallization starts early, the crystals have more time to grow and increase overall crystallinity. This increase in the crystallization confirmed the improvement in the mechanical properties of GNP-based PA66 nanocomposites. Further crystallinity investigation can be conducted using the equation below:

(8) $$X_{C}=\left(\frac{\nabla H_{M}}{\nabla H_{M}^{100\%}}\right)\times 100$$

where *X_C_* is the degree of crystallization, Δ*H_M_* is the melting enthalpy and $\nabla H_{M}^{100\%}$ is the melting enthalpy of 100% crystalline PA66. Neat crystalline PA66 has a melting enthalpy of 188.4 J/g [55]. Based on this equation, crystallinity degrees of neat PA66 and PA66-GNP nanocomposites have been calculated and given in Table 4.

**Figure 4:**
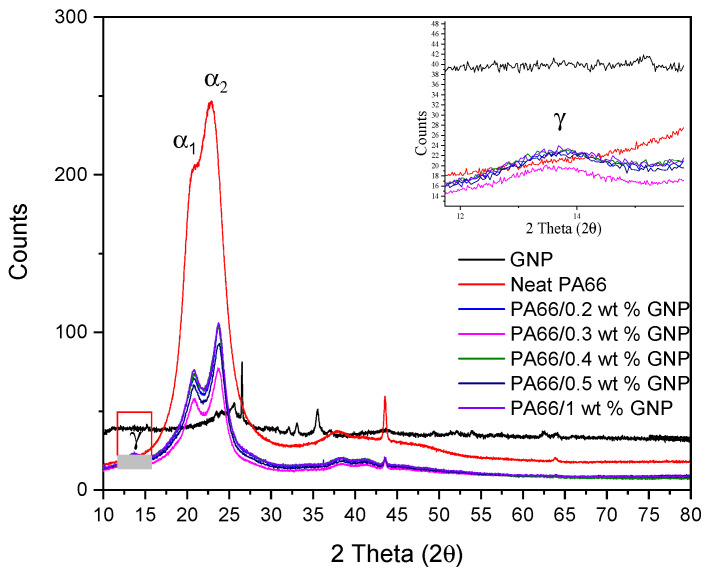
Diffraction patterns of neat PA66 and PA66/GNP nanocomposites after elongation at break.

XRD also investigated crystalline properties of neat PA66 and its nanocomposites to validate GNP’s nucleation effect and understand its impact on crystallinity. XRD studies were carried out with the test specimens obtained after the tensile test. Figure 4 shows XRD patterns of neat PA66 and its nanocomposites with different GNP loadings. PA66 shows two characteristic peaks as α_1_ and α_2_ around 2θ = 20° and 2θ = 24° degrees corresponding to (100) and (010)/(110) overlapping peaks, respectively [56]. With GNP incorporation into the matrix, another peak indicating γ (002) phase around 2θ=14° arose, which was previously thermodynamically unstable at room temperature for the neat polymer [57]. The mismatch between crystallization ratios obtained from DSC and XRD results stems from extreme strain shown by the neat PA66, and this strain results in distortion of the lattice and change in d-spacings [58]. GNP reinforced PA66 composites did not show such strain. Consequently, the resulting mismatch was significantly lower. The discrepancy in crystallinity values from DSC and XRD values is also reported in the literature [59]. The intercalation of GNP between the polymer matrix is indicated by the separation of α_1_ and α_2_ peaks. The main peak positions of α_1_ and α_2_ as well as their ratio, are given in Table 5. Crystallinities of the samples have been calculated by the software given in Table 6.

### 3.3. The Effect of GNP as a Reinforcement on the Rheological Behaviour of PA66 Nanocomposites

**Figure 5:**
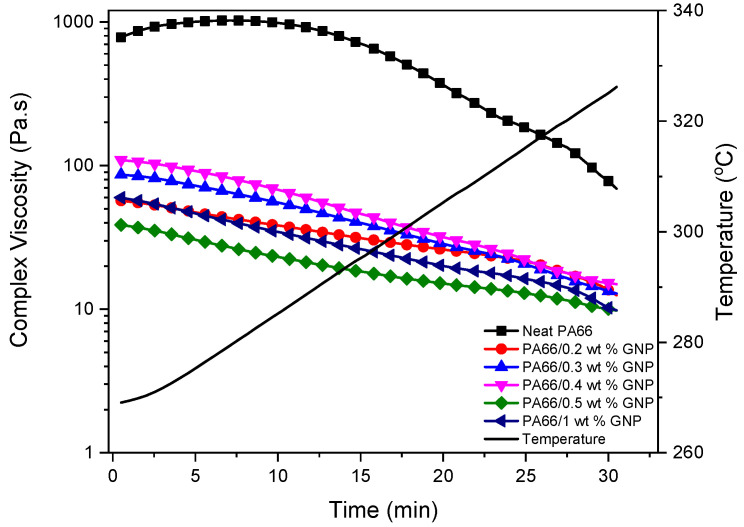
Change in complex viscosity with the addition of GNP to the PA66 as a function of time and temperature.

**Figure 6:**
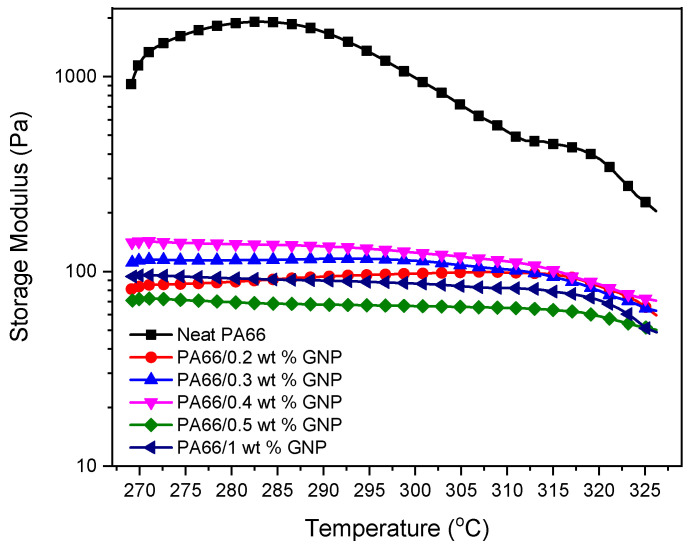
Storage Moduli of neat PA66 and PA66 GNP composites as a function of temperature.

Complex viscosity is an essential parameter for estimating the processability of thermoplastic composites [60]. As the temperature gets close to the polymer’s melting point, complex viscosity drops for both neat PA66 and GNP-PA66 composites. It can be speculated that the long-distance movement of the polymer chains is limited, which is indicated by reaching a stable viscosity value at higher temperatures [61]. Rheological behavior regarding the complex viscosity (η*) and storage moduli of the neat PA66 and PA66-GNP composites using temperature sweep method with constant frequency have been given in Figure 5 and Figure 6, respectively. The addition of GNP to the polymer matrix changes the viscosity of the composites, which affects the storage modulus significantly [62]. The storage modulus demonstrates the elasticity portion of the viscoelastic behavior, and it indicates the energy stored before permanent deformation. Decreasing storage modulus at high temperatures shows that the elastic part of the composites, which is the polymer itself, melts and resembles a typical liquid’s behavior. High storage moduli of 0.3% and 0.4% GNP loaded PA66 composites match the composites’ tensile and flexural properties.

**Figure 7:**
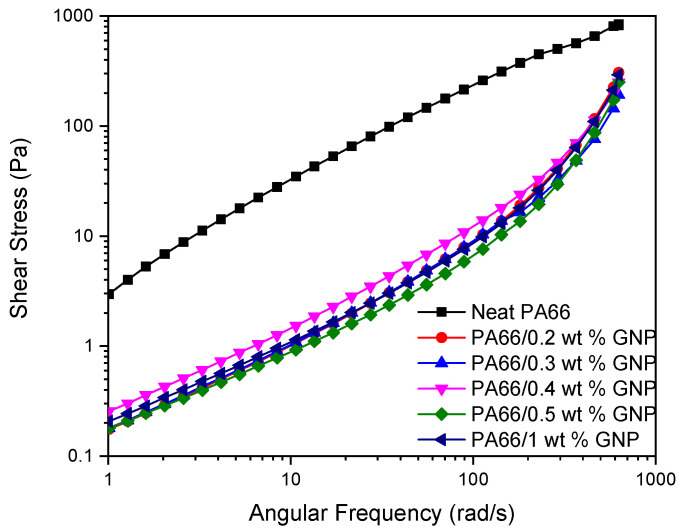
Change in the shear stress of PA66 and its GNP-based nanocomposites with the angular frequency.

**Figure 8:**
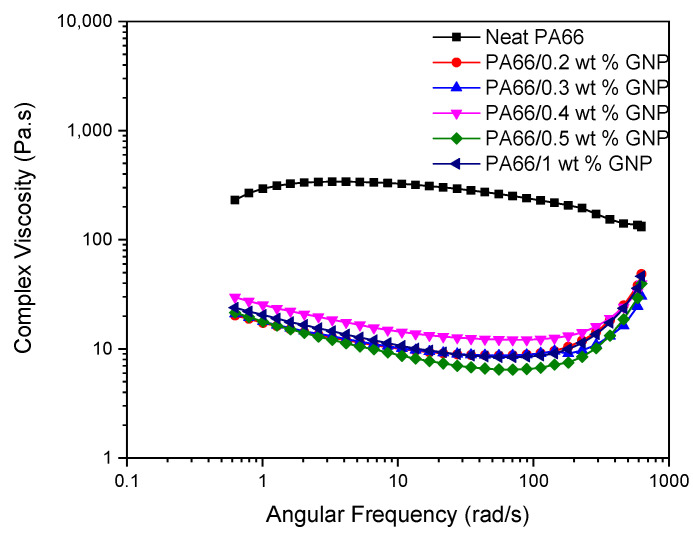
Complex viscosity of neat PA66 and its GNP-based nanocomposites as a function of angular frequency.

**Figure 9:**
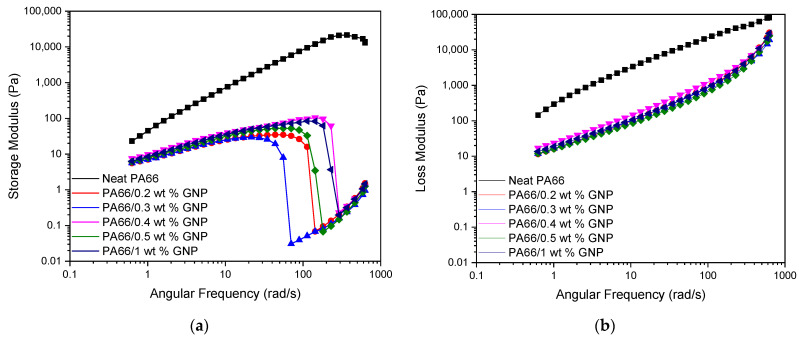
Change in storage moduli (**a**) and loss moduli (**b**) of neat PA66 and PA66/GNP nanocomposites.

Investigating the rheological behavior of the PA66 and its GNP-based nanocomposites at the melt temperature (280 °C) as a function of angular frequency gives insight into the injection molding process dynamics dispersion of the GNP in the PA66 matrix. During the injection molding, the flow rates at the center of the mold and the outer layers are different, and this creates a shearing effect on the polymer. Higher shear stresses can cause molecules to break and the loss of mechanical properties. Figure 7 shows the shear stresses of PA66 and PA66/GNP nanocomposites as a function of angular frequency. Here, GNP’s addition leads to a decrease in the shear stress, which is confirmed by the increase in the mechanical properties. Figure 8 displays the complex viscosity of the samples. It is seen that both in temperature and frequency sweep, the polymer’s viscosity decreases with the addition of GNPs. This behavior resembles the shear-thinning phenomena, and the exfoliation of GNPs can explain the mechanism behind it during the process aiding the slip between the polymer matrix and GNPs [63]. Furthermore, this decrease in the viscosity improves the melt processability of the polymer. Figure 9 displays the storage (a) and loss (b) moduli of the samples. As is expected, storage moduli and loss moduli for all samples increase up to a particular frequency. However, at higher frequencies around 100 rad/s, the storage moduli show a significant decrease for GNP-based PA66 composites while loss moduli increase. Thus, implying rigid graphene particles breaking the polymer chains at high frequencies results in low energy storage.

### 3.4. Numerical Simulation and Modeling of GNP Orientation State

**Figure 10:**
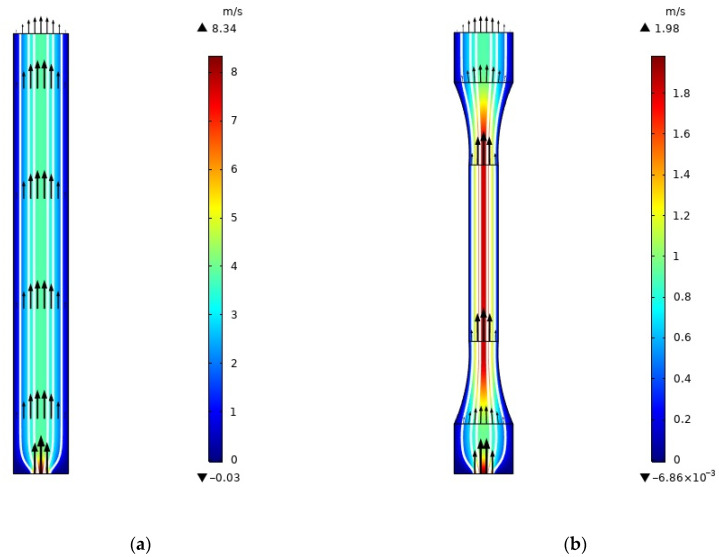
The vertical component of the velocity field (i.e., v) and velocity profiles for (**a**) rectangular and (**b**) dog bone samples. The white lines represent velocity streamlines.

To attain further insight into the GNP ratios, mold shape, and their effects on the filling process in an air-filled enclosure, numerical simulations for six various cases are accomplished. The fluid domain is designed based on standard tensile geometry (ISO 527-2 1BA) and bending test (ISO 178) samples. The maximum pressure is assigned as $P_{max}=1.9\;\mathrm{MPa}$ at the inlet boundary, which is obtained from the experimental procedure. As the simulations are performed with the COMSOL Multiphysics software as it is provided in the above sections, the vertical component of the velocity field (i.e., v). and velocity profiles for PA66 with 0.3 wt% GNP for rectangular and dog bone samples are illustrated in Figure 10.

**Figure 11:**
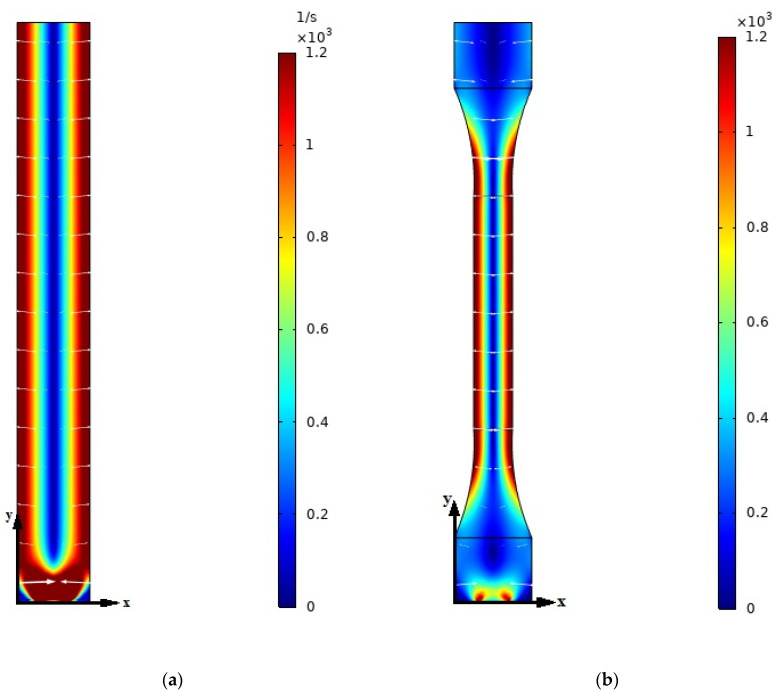
Strain rate and average GNP orientation for PA66 with 0.3 wt% GNP for (**a**) rectangular and (**b**) dog bone sample. Arrows represent the average GNPs’ axis orientation.

In Figure 11, the background color represents the shear rate distribution in the injection domain. The average GNPs’ axis orientation is shown by two perpendicular axes whose magnitude and angles were calculated from eigenvalues and eigenvectors of the second-order tensor orientation $a_{2}$. As shown in these plots, the shear rate value is high for the close to the wall areas leading to have strong eigenvalues in these domains, while in the areas with the low shear rate, the prediction of the orientation is weak, and GNPs are oriented randomly in the flow domain. As Folgar–Tucker model suggests, allocating nonzero value to $C_{I}$ leads to randomly distributing the particles in the flow domain, causing different orientations along the streamlines [47]. It is worth mentioning here that since the GNPs are usually treated as 2-dimensional disk shape particles [64], the amount of λ in the Equation (6) is equal to −1 and the direction of $\vec{p}$ vector in Equation (4) represents the main axis of the particle which is perpendicular to the surface of the GNPs [35]. Therefore, the arrow shown in the Figure 11 is the direction of the $\vec{p}$ vector.

**Figure 12:**
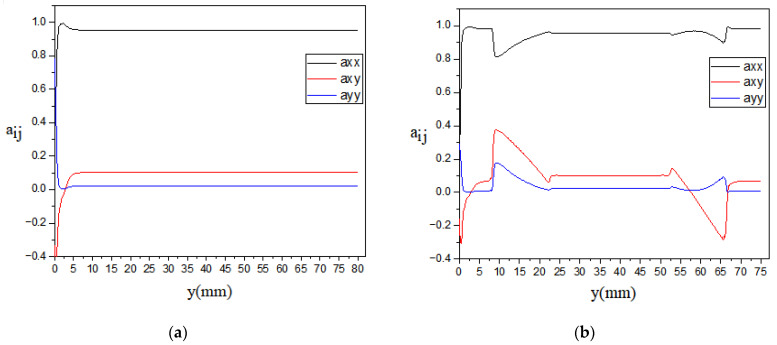
Evolution of second-order orientation tensor components ($a_{xx},a_{xy}a_{yy})$ along with a streamline very close to the vertical wall (**a**) for rectangular and (**b**) dog bone samples.

**Figure 13:**
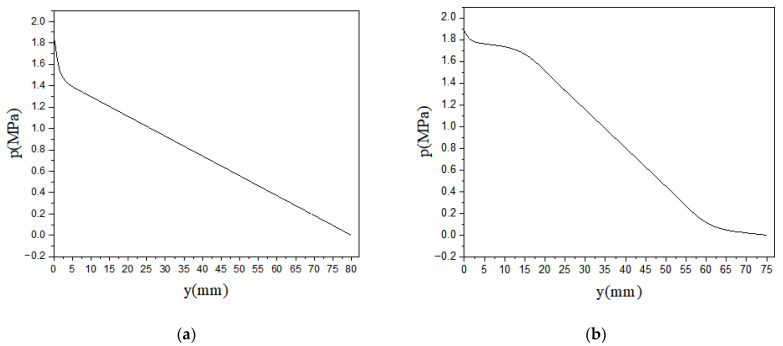
Pressure drop along x = 5mm (**a**) for rectangular and (**b**) dog bone samples.

The GNPs orientation was explained by evaluating the variation in orientation tensor components along the flow field, as shown in Figure 12. Dog bone sample yielded an oscillation in GNPs orientation along the flow direction compared to the rectangular domain where GNP orientation has an almost constant behavior during the injection process. According to Figure 12b, it can be seen that the GNPs orientation along the flow direction decreases in the convergent area in the dog bone domain, where it causes an increase in the GNPs’ alignment in the two other axis directions. For the area between the y = 22.5 to 52.5 mm in the dog bone domain, where the velocity profile and shear rate values remain constant, the orientation tensor components do not undergo significant changes. The pressure drop is examined along the symmetrical axis (at x = 5 mm) and shown in Figure 13 for rectangular and dog bone samples. The pressure drops from 1.9 MPa in the inlet to 0 at the outlet boundary, as it has been defined. While the pressure drop has a constant slope in the domains with a fixed cross-section, the pressure drop exhibits a slightly constant slope at the dog bone shape’s divergent and convergent areas. This phenomenon can affect the GNPs orientation in these areas, as has been reported in the previous studies [65].

**Figure 14:**
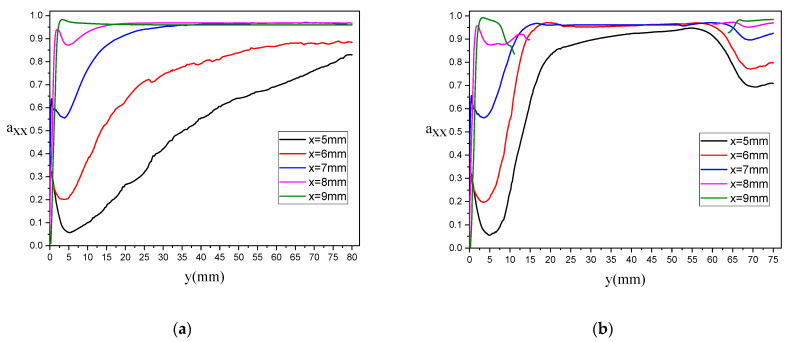
$a_{xx}$ components along some streamlines/vertical lines parallel to the symmetry axis for PA66 with 0.3 wt% GNP: (**a**) for rectangular and (**b**) dog bone sample.

The GNPs orientation distribution is also examined along five streamlines (x = 5,6,7,8, and 9 mm), as shown in Figure 14. The discontinuous lines in Figure 14b are due to the absence of the streamlines in the longitudinal axes in the dog bone sample. For rectangular sample and along the symmetrical axis at x = 5 mm, $a_{xx}$ decreases from isotropic orientation state in front of the injection entrance, and then it increases and becomes equal to 0.83 at the end of the path, as illustrated in Figure 14a. As the x approaches the wall at the rectangular domain, $a_{xx}$ increases, where it can be said that for x = 9 mm, the particles are entirely oriented along the flow direction. For the dog bone sample, Figure 14b, the evolution of $a_{xx}$ is different than one observed for the rectangular sample. Along the symmetrical axis at x = 5 mm, $a_{xx}$ decreases from isotropic orientation state in front of the injection entrance, and then it increases until the flow arrives in the domains with a fixed cross-section. The slope of $a_{xx}$ profiles remain almost constant in this domain, especially for streamlines near the no-slip boundary, and finally, with entering the flow in the divergence domain, the value of $a_{xx}$ drops from 0.95 to 0.71.

**Figure 15:**
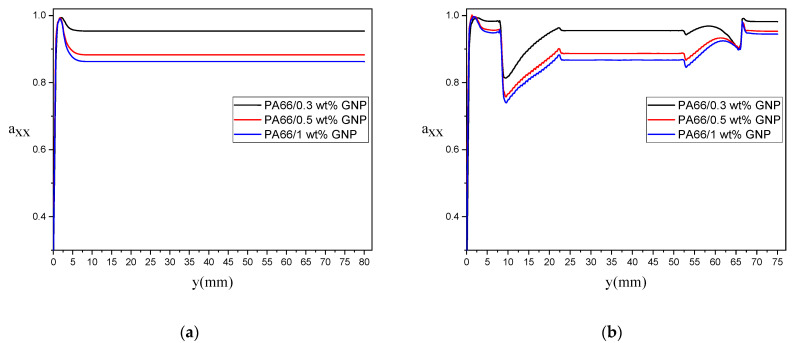
Evolution of $a_{xx}$ along with a streamline very close to the vertical wall for different GNPs wt%: (**a**) for rectangular and (**b**) dog bone samples.

**Figure 16:**
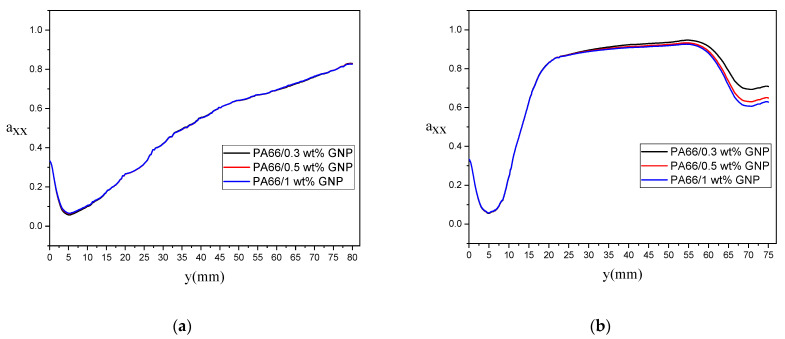
Evolution of $a_{xx}$ along the central streamline (symmetry line) for different GNPs wt%: (**a**) for rectangular and (**b**) dog bone samples.

The effect of different GNPs loadings on the evolution of the first component of second-order orientation tensor ($i.e.,\;a_{xx}$) are represented in Figure 15 and Figure 16. In Figure 15, $a_{xx}$ evolution along a streamline very close to the vertical wall is provided. The direction of the $\vec{p}$ vector almost stands in the x-direction for the rectangular samples, which means that the GNPs are oriented in the samples’ longitudinal direction. According to Figure 15a, the bending sample with lower GNP loading, such as 0.3 wt%, has the most alignment in the x-direction with $a_{xx}=0.95$, while increasing the GNP loading causing a decrease in the $a_{xx}$, as this component value decreases to 0.86 for 1.0% GNP. The alignment of GNPs for the dogbone sample is shown in Figure 15b, where the GNPs orientation from the longitudinal direction drops sharply by entering the convergence zone of the dogbone, and then, due to the growth of velocity gradients, it increases in the straight portion of the dogbone and remains at $a_{xx}=0.95$ for 0.3 wt% GNP and $a_{xx}=0.866$ for 1.0 wt% GNP. At the entrance region of the dogbone’s divergence zone, it shows another rise at the end of the domain. The orientation of GNPs along the symmetry line is represented in Figure 16. The change of GNP loading in the rectangular sample does not significantly affect the orientation of particles on the central line since the lack of velocity gradients in this region. On the other hand, the average value of $a_{xx}$ at the steady-state after the convergence zone in the dogbone sample increases up to $a_{xx}=0.95$ for 0.3% GNP, which defines a high alignment of GNPs in the flow direction. This value decreases in the expansion zone at the end of the dogbone, causing a rise of $a_{yy}$, which means that GNPs will rotate around their main axis.

According to Figure 15 and Figure 16, it can be concluded that GNPs alignment is increasing for streamlines near the wall as the same behavior is found in previous papers in the literature [47,49,66]. Also, a matrix with a lower GNP additive shows more alignment of particles along the flow direction. Therefore, the great improvement of tensile/flexural properties of PA66/GNP nanocomposites for the PA66/0.3 wt% GNP can be related to the alignment of reinforcements in the domain.

### 3.5. Cross-Sectional Analysis of GNP Reinforced PA66 Nanocomposites

**Figure 17:**
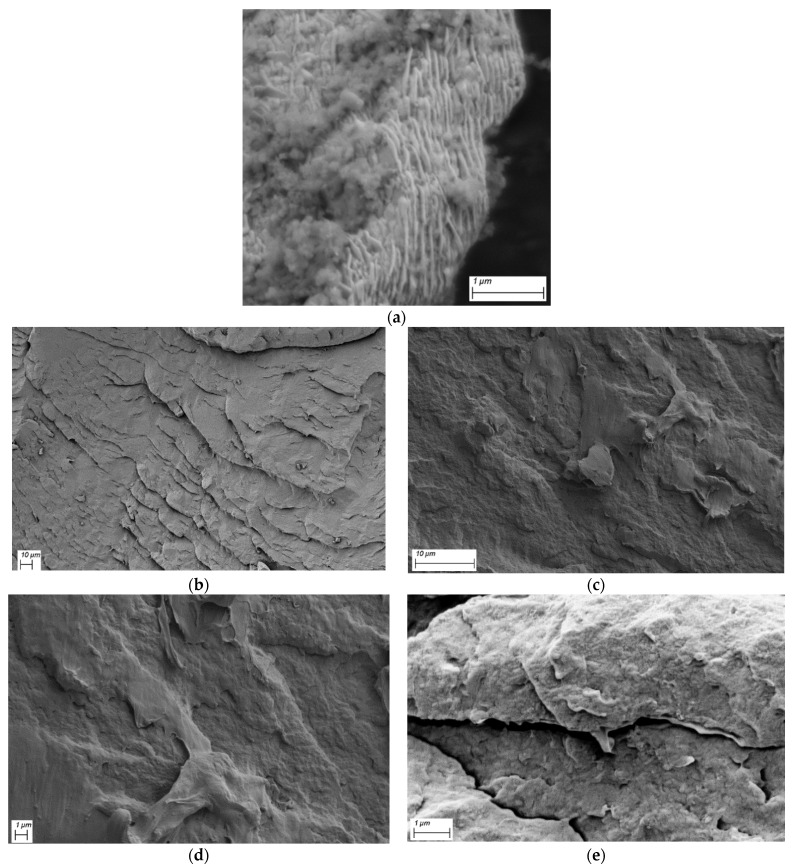
Platelet structure of (**a**) GNP, and Freeze-fractured surfaces of (**b**) neat PA66, (**c**) and (**d**) 0.4 wt% GNP loaded PA66 nanocomposite at different magnifications, and (**e**) 0.2 wt% GNP/PA66 nanocomposite.

Scanning electron microscopy (SEM) is an important technique to analyze the surface morphology of GNPs, neat PA66, and GNP reinforced PA66 nanocomposites. The surface topology of the GNPs, as well as, cross-sectional fracture surfaces of freeze-fractured test specimens were investigated by SEM, and the distribution of graphene nanoplatelets in the polymer matrix is observed. This is also a useful technique to observe the distribution of and compare the samples’ fracture surfaces. Figure 17a demonstrates the well-defined platelet structure of the waste tire-derived graphene. Figure 17b displays the freeze-fractured surface of neat PA66, while Figure 17c shows the freeze-fractured surface of 0.4 wt% GNP/PA66 nanocomposite. The surface of the GNP-based nanocomposite is smoother compared to the neat counterpart. In addition, the fragmented surface on the neat polymer is observed; however, with the addition of GNP, a film-like coating is observed on the surface, preventing the fragmented fracture. Figure 17 also exhibits the freeze-fracture surface of the 0.4 wt% GNP/PA66 (d), and 0.2 wt% GNP/PA66 (e). The fragmented surface is still visible at the 0.2 wt% loadings; however, a film-like structure is also obtained. Additional images of the nanocomposites at different loadings have been given in Figure S5 (see supporting information).

## 4. Conclusions

In the present study, graphene from waste tire was successfully distributed in PA66 matrix by thermokinetic mixer at high shear rates with the loadings less than 1 wt%. This study, experimentally investigating the effect of different GNP loading, proposes a multidisciplinary approach along with the numerical validation to understand the performance of the GNP reinforced nanocomposites. GNP agglomeration issue during thermoplastic processing was resolved by providing reinforcement/matrix interfacial strength. It was observed that surface oxygen functional groups on GNP surface provided ease intercalation through PA66 polymer chains during melt-phase. The numerical validation study via finite element analysis was conducted to confirm an ideal GNP orientation state on the effect of fluid domain, its geometry, and filler concentration. The laminar flow equations, besides appropriate boundary conditions, were also solved, and then the velocity gradients at every spatial position were applied to calculate PDE equations of the GNPs orientation. With this study, the incorporation of GNP in PA66 increased the crystallization temperature since it performed as both nucleating and reinforcing agents. The optimum loading percentage was determined as 0.3 wt% GNP, resulting in 30% and 42% increase in tensile strength and modulus, respectively, compared to neat PA66 matrix. Similarly, an increase of 20% and 43% in flexural strength and modulus with 0.3 wt% loading was recorded, respectively. The process governed by the rheology of nanocomposites was also analyzed for the adaptation of numerical investigation to model the fluid flow during injection. Overall, waste tire-derived graphene is a promising reinforcement material, and at lower loadings, significant mechanical property improvements are obtained. Consequently, this comprehensive and interdisciplinary study will fasten the development of scalable technologies for graphene compounding by conventional extrusion and injection processes. Furthermore, the conversion of waste materials into a value-added material will contribute to circular economy issues and lower the cost of graphene by using waste tires as a starting material and applying recycling and upcycling technologies for mass production in the plastic industry.

## Figures and Tables

**Table 1:** Tensile properties and improvements of neat PA66 and PA66/GNP nanocomposites.

Sample	Tensile Strength (MPa)	Improvement (%)	Tensile Modulus (MPa)	Improvement (%)	Tensile Strain at Break (%)
Neat PA66	54.9 ± 6	-	2334 ± 154	-	16.8 ± 21
PA66/0.2 wt% GNP	56.4 ± 10	2.7	3128 ± 150	34.0	2.8 ± 1.2
PA66/0.3 wt% GNP	71.6 ± 1	30.4	3306 ± 50	41.7	3.5 ± 0.2
PA66/0.4 wt% GNP	60.5 ± 1	10.2	3350 ± 143	43.5	2.6 ± 0.1
PA66/0.5 wt% GNP	60.2 ± 4	9.7	3080 ± 64	32.0	2.8 ± 0.1
PA66/1.0 wt% GNP	56.2 ± 12	2.4	3160 ± 513	35.4	2.7 ± 0.4

**Table 2:** Improvement percentages of neat PA66 and PA66/GNP nanocomposites in flexural properties.

Sample	Flexural Strength (MPa)	Improvement (%)	Flexural Modulus (MPa)	Improvement (%)	Flexural Strain (%)
Neat PA66	93.1 ± 2	-	2250.0 ± 68	-	7.05 ± 0.3
PA66/0.2 wt% GNP	83.8 ± 9	−9.9	3170.0 ± 167	40.8	2.79 ± 0.4
PA66/0.3 wt% GNP	113.0 ± 6	21.3	3210.0 ± 100	42.6	4.72 ± 1.4
PA66/0.4 wt% GNP	114.0 ± 10	22.4	3160.0 ± 40	40.4	5.15 ± 1.5
PA66/0.5 wt% GNP	96.30 ± 7	3.4	3220.0 ± 41	43.1	3.25 ± 0.4
PA66/1.0 wt% GNP	108.0 ± 21	16.0	3230.0 ± 54	43.5	4.84 ± 2.0

**Table 3:** Melting and crystallization parameters of neat PA66 and PA66/GNP nanocomposites.

Sample	Melting Onset Temperature (°C)	Melting Peak Temperature (°C)	Melting Integral, ΔH_m_ (J/g)	Crystallization Onset Temperature (°C)	Crystallization Peak Temperature (°C)	Crystallization Integral, ΔH_c_ (J/g)
Neat PA66	250	262	−64.18	219	209	56.56
PA66 + 0.2 wt% GNP	250	260	−83.17	241	238	63.33
PA66 + 0.3 wt% GNP	251	262	−75.25	241	237	57.15
PA66 + 0.4 wt% GNP	250	262	−74.62	241	237	56.56
PA66 + 0.5 wt% GNP	250	262	−68.66	241	237	51.57
PA66 + 1.0 wt% GNP	250	260	−78.21	243	239	59.09

**Table 4:** Crystallinity degrees of neat PA66 and its GNP-based nanocomposites obtained from DSC characterization.

Sample	Crystallinity (%)	Amorphous (%)
Neat PA66	34.06	65.94
PA66 + 0.2 wt% GNP	44.14	55.86
PA66 + 0.3 wt% GNP	39.94	60.06
PA66 + 0.4 wt% GNP	39.60	60.40
PA66 + 0.5 wt% GNP	36.44	63.56
PA66 + 1.0 wt% GNP	41.51	58.49

**Table 5:** α_1_ and α_2_ peak positions of neat PA66 and its GNP-based nanocomposites.

Sample	α_1_ Position	α_2_ Position	α_1 /_ α_2_ Ratio
Neat PA66	20.86	22.93	0.90
PA66 + 0.2 wt% GNP	20.72	23.84	0.86
PA66 + 0.3 wt% GNP	20.90	23.66	0.88
PA66 + 0.4 wt% GNP	20.84	23.56	0.88
PA66 + 0.5 wt% GNP	20.76	23.84	0.86
PA66 + 1.0 wt% GNP	20.88	23.72	0.88

**Table 6:** Crystallinity degrees of neat PA66 and GNP/PA66 nanocomposites were calculated by the software.

Sample	Crystallinity (%)	Amorphous (%)
Neat PA66	51.0	49.0
PA66 + 0.2 wt% GNP	35.4	64.6
PA66 + 0.3 wt% GNP	34.8	65.2
PA66 + 0.4 wt% GNP	35.0	65.0
PA66 + 0.5 wt% GNP	35.9	64.1
PA66 + 1.0 wt% GNP	35.9	64.1

## Data Availability

The data presented in this study are available on request from the corresponding author.

## Supplementary Materials

The following are available online at https://www.mdpi.com/2073-4360/13/6/949/s1, Figure S1: (a) XPS survey scan spectrum, (b) Raman spectrum, and (c) TEM image of GNPs, Figure S2: X-ray Diffraction (XRD) spectrum of GNP, Figure S3: 2D mesh for standard bending test and tensile test samples, Figure S4: Time series of the a*_xx_* across the longitudinal sidewall of the domain using different (a)time steps and (b) mesh size, Figure S5: Freeze-fracture surfaces of PA66/GNP nanocomposites at 0.3 wt% GNP (a), 0.5 wt% GNP (b), and 1 wt% GNP loadings (c).
