# Supplementary material for: Experimental and Numerical Investigation of Flow and Alignment Behavior of Waste Tire-Derived Graphene Nanoplatelets in PA66 Matrix during Melt-Mixing and Injection
Source: Polymers (Basel). 2021 Mar 19;13(6):949. doi: 10.3390/polym13060949 (PMC8003376; doi:10.3390/polym13060949)
Supplement: Supplementary file 1 [file polymers-13-00949-s001.pdf]

polymers

Research article

Experimental and Numerical Investigation of flow and alignment behavior of Waste Tire-derived Graphene Nanoplatelets in PA66 matrix during melt-mixing and injection

Kuray Dericiler [1,2], Hadi Mohammadjafari Sadeghi [1,2], Yavuz Emre Yagci [3], Hatice S. Sas [1,2,*] and Burcu Saner Okan [1,2,*]

[1] Integrated Manufacturing Technologies Research and Application Center & Composite Technologies Center of Excellence, Manufacturing Technologies, Sabanci University, Istanbul 34906, Turkey; kuraydericiler@sabanciuniv.edu (K.D.); mhadi@sabanciuniv.edu (H.M.S.)
[2] Faculty of Engineering and Natural Sciences, Sabanci University, Tuzla, Istanbul 34956, Turkey
[3] Farplas Otomotiv A.S., Taysad Organize Sanayi Bölgesi (TOSB), Kocaeli 41420, e.yagci@farplas.com (Y.E.Y.)
* Correspondence: bsanerokan@sabanciuniv.edu, haticesas@sabanciuniv.edu

In this study, GNPs derived from waste tire recycle and upcycle process have been used as a reinforcing agent for polyamide 66. X-ray Photoelectron Spectroscopy (XPS) shows that the GNPs contain 87 wt% carbon and 9.1 wt% oxygen containing surface functional groups (Fig S1(a)). Characteristic graphene peaks of D and G are located around 1358 and 1580 cm-1 (Fig S1(b)). TEM image in Fig S1(c) indicates that the platelet formations have an average length of 50±4 nm.

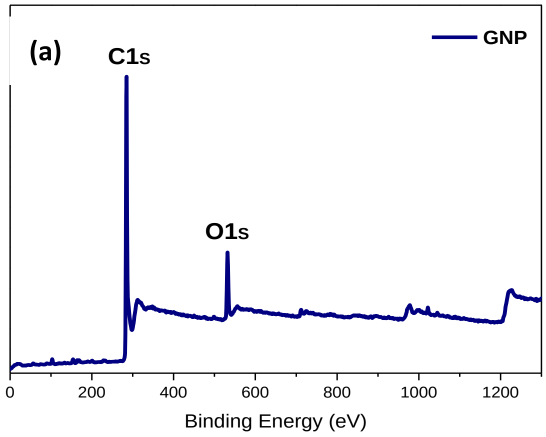

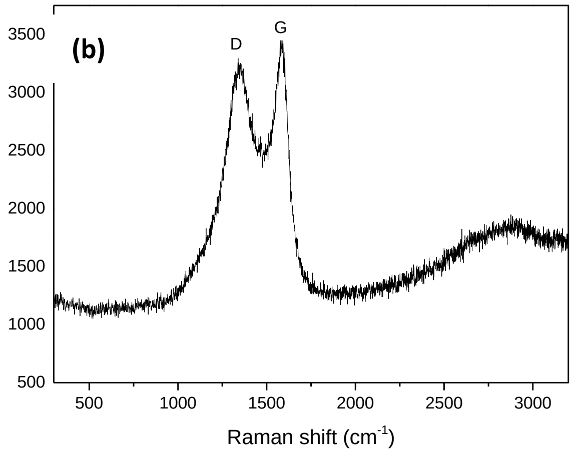

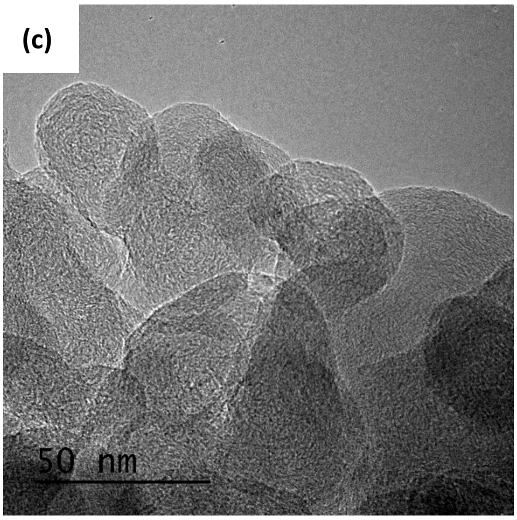

Figure S1. (a) XPS survey scan spectrum, (b) Raman spectrum, and (c) TEM image of GNP

X-ray Diffraction (XRD) spectrum of graphene nanoplatelets in Figure S2 indicates a crystallinity degree of 24.1%.

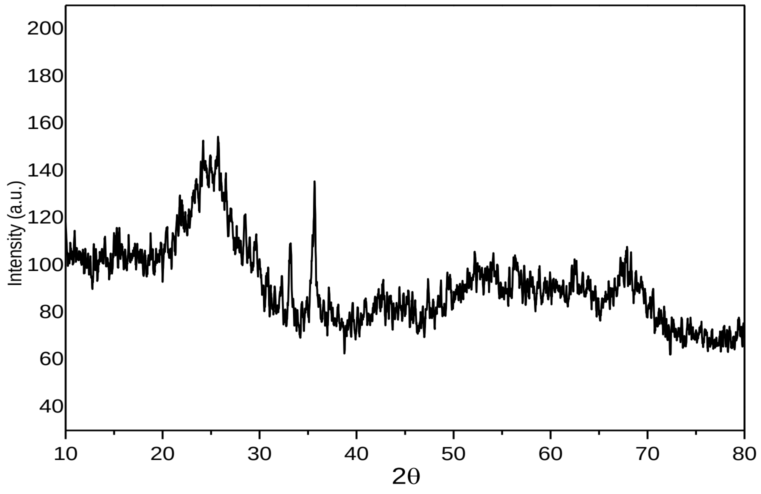

Figure S2. X-ray Diffraction (XRD) spectrum of GNP

Figure S3 demonstrates the generated 2d mesh by COMSOL software for two types of the flow domain:

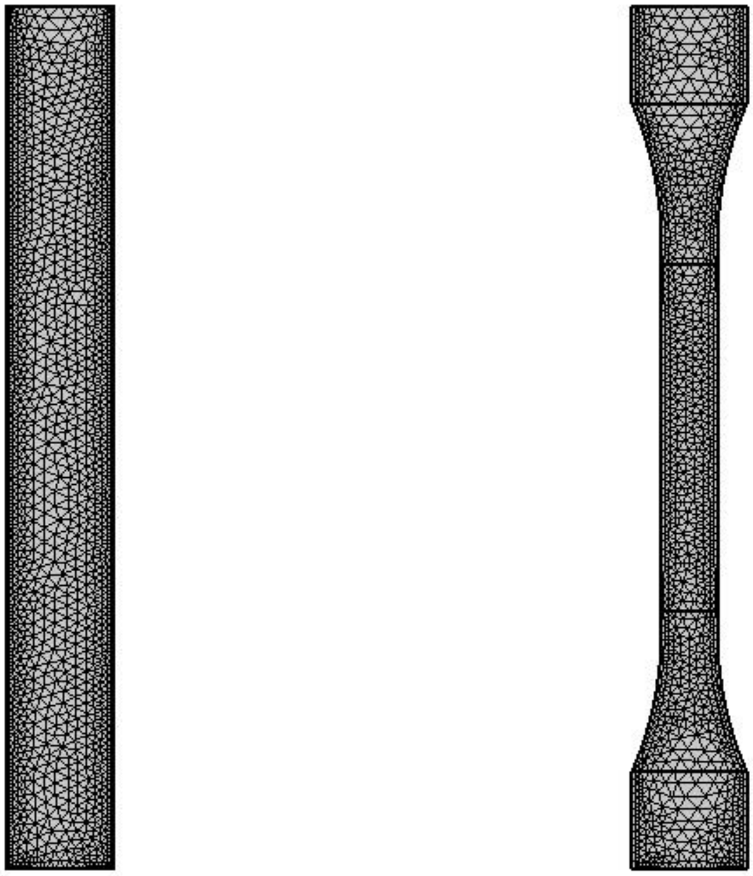

Figure S3. 2D mesh for standard bending test and tensile test samples

A heterogeneous triangular grid is applied to divide the whole cavity into finite elements. While this segmentation is large in the middle of the domain, the grid is partially fine in the surrounding boundaries and injection location.

In order to obtain a solution that is independent of time and mesh size, the numerical procedure was performed for three various time steps and mesh sizes. For this means, a rectangular domain was selected, and the time series of the a_{xx} across the longitudinal sidewall of the domain is presented in Figure S4.

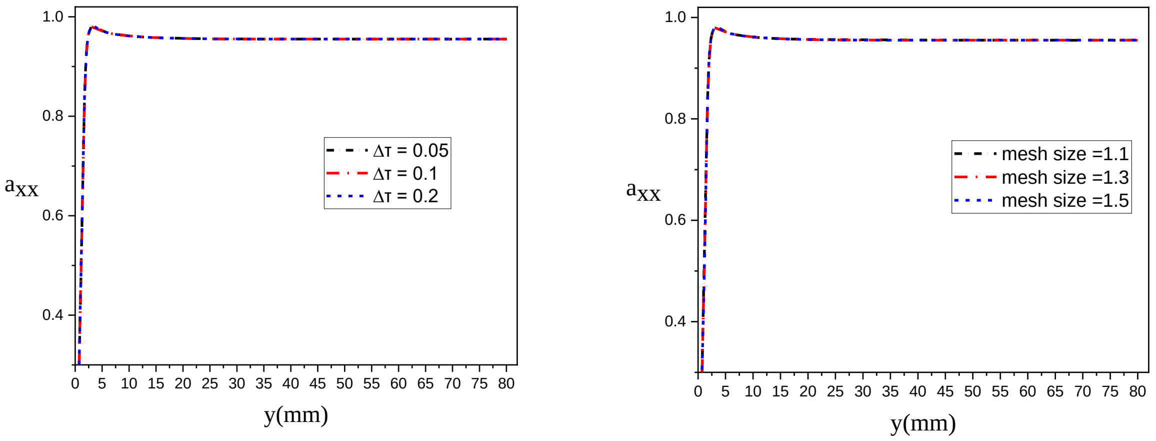

Figure S4. Time series of the a_{xx} across the longitudinal sidewall of the domain using different (a)time steps and (b) mesh size.

According to this figure, there is no significant grid and time sensitivity for the selected number of elements and time steps. Also, the calculated error between the three configurations is small. So, each of the following tests leads to satisfactory results; however, to reduce computing time, the system with the time step Δτ = 0.1 second and mesh size with maximum element size 1.1 was selected for the following analysis.

Film-like formations on the PA66 surface is visible for different GNP loadings were visible on the freeze-fractured surfaces of the nanocomposites. Figure S5 displays the freeze-fractured surfaces of the PA66/GNP nanocomposites at 0.3 wt% GNP (a), 0.5 wt% GNP (b), and 1 wt% GNP loadings (c).

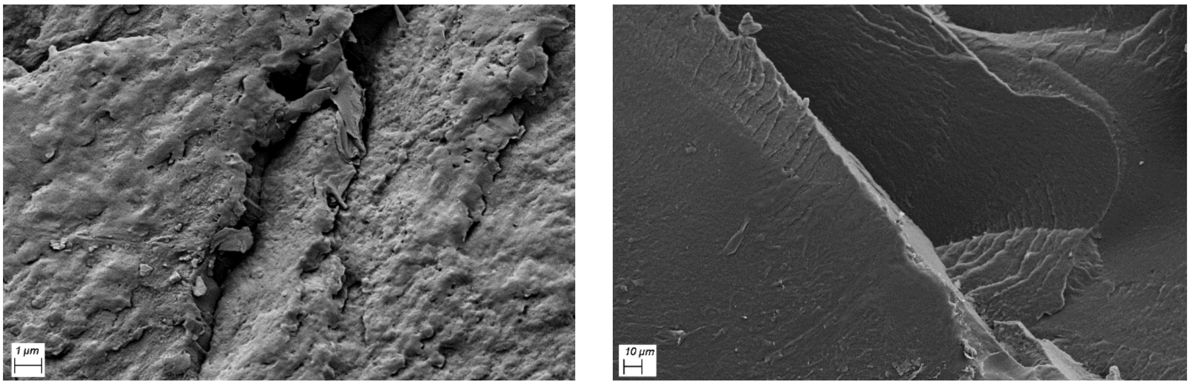

(a) (b)

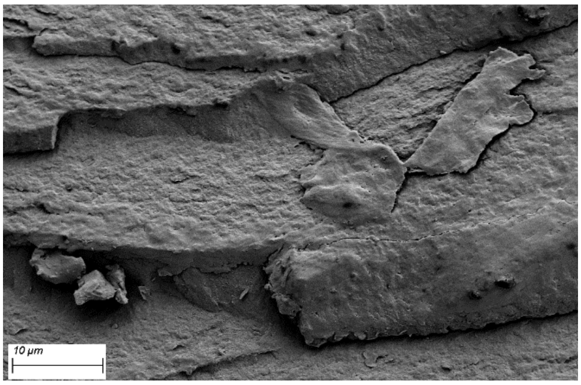

(c)

Figure S5: Freeze-fracture surfaces of PA66/GNP nanocomposites at 0.3 wt% GNP (a), 0.5 wt% GNP (b), and 1 wt% GNP loadings (c).
